# Chronic Lower Limb Pain Unveiling a Rare Case of Spindle Cell Soft Tissue Sarcoma: A Diagnostic Odyssey

**DOI:** 10.7759/cureus.48634

**Published:** 2023-11-10

**Authors:** Shreya Khandelwal, Pratap Parihar, Rajasbala Dhande, Anshul Sood

**Affiliations:** 1 Radiodiagnosis, Jawaharlal Nehru Medical College, Datta Meghe Institute of Higher Education and Research, Wardha, IND

**Keywords:** tumors, ct, mri, spindle cell soft tissue sarcomas, limb pain

## Abstract

Spindle cell soft tissue sarcomas are rare and challenging malignancies that tend to masquerade as benign conditions. This case report presents a 70-year-old female who sought medical attention due to persistent right lower limb pain and swelling over one year. Despite prior consultations at private clinics, her symptoms continued to progress. A tender, palpable swelling was noted upon examination, prompting further diagnostic investigations. Initial X-ray results yielded inconclusive findings, necessitating an MRI study with contrast. The MRI unveiled a substantial multi-lobulated spindle-shaped mass lesion exhibiting heterogeneous enhancement and altered signal intensity, measuring 7.3 x 2.5 x 2.2 centimeters. Additional nodular lesions in the periarticular region posterior to the ankle joint confirmed the diagnostic suspicion of spindle cell sarcoma, supported by orthopedic evaluation. Symptomatic management was initiated with analgesics and antibiotics, alongside a recommendation for biopsy. Histopathological examination of the biopsy specimen confirmed the presence of spindle cell soft tissue sarcoma under high magnification. This case underscores the diagnostic challenges of spindle cell sarcomas and the imperative role of a multidisciplinary approach in their accurate diagnosis and management.

## Introduction

Spindle cell soft tissue sarcomas represent a diverse group of rare malignancies originating from mesenchymal tissues, often characterized by spindle-shaped cells [1]. These neoplasms frequently pose diagnostic challenges due to their clinical variability and nonspecific presentations [2]. Over the years, extensive research has contributed to our understanding of these tumors' clinical characteristics, pathological features, and management. This introduction provides an overview of spindle cell sarcomas, highlighting the importance of early diagnosis and comprehensive interdisciplinary management [3].

Spindle cell sarcomas can arise in various anatomic locations, further complicating their diagnosis and treatment [4]. The clinical presentation of spindle cell sarcomas often involves non-specific symptoms such as pain and swelling, making it crucial for physicians to consider malignancy in the differential diagnosis [5]. Imaging techniques, particularly MRI and CT, have proven valuable in assessing the extent and characteristics of these tumors [6].

Histopathological examination remains the cornerstone of spindle cell sarcoma diagnosis, with immunohistochemistry playing a vital role in distinguishing subtypes [7]. The utility of specific markers, such as CD34, S-100, and desmin, has been well documented in the literature [8]. Moreover, molecular and genetic studies have unveiled significant insights into the pathogenesis of spindle cell sarcomas, aiding in diagnosis and potential targeted therapies [9].

Treatment strategies for spindle cell sarcomas typically involve surgical resection, often complemented by radiation therapy or chemotherapy, depending on the tumor's grade and location [10]. A multidisciplinary approach incorporating orthopedicians, radiologists, pathologists, and oncologists is pivotal in achieving optimal patient outcomes [11].

## Case presentation

A 70-year-old woman was brought to the outpatient department of a tertiary care hospital by her son, presenting with a one-year history of pain in her right lower limb and associated swelling. An in-depth review of the patient's medical history revealed progressively worsening pain and swelling over the past year. During this time, she sought treatment from private clinics, where her symptoms were managed with analgesics and calcium supplements.

**Figure 1:**
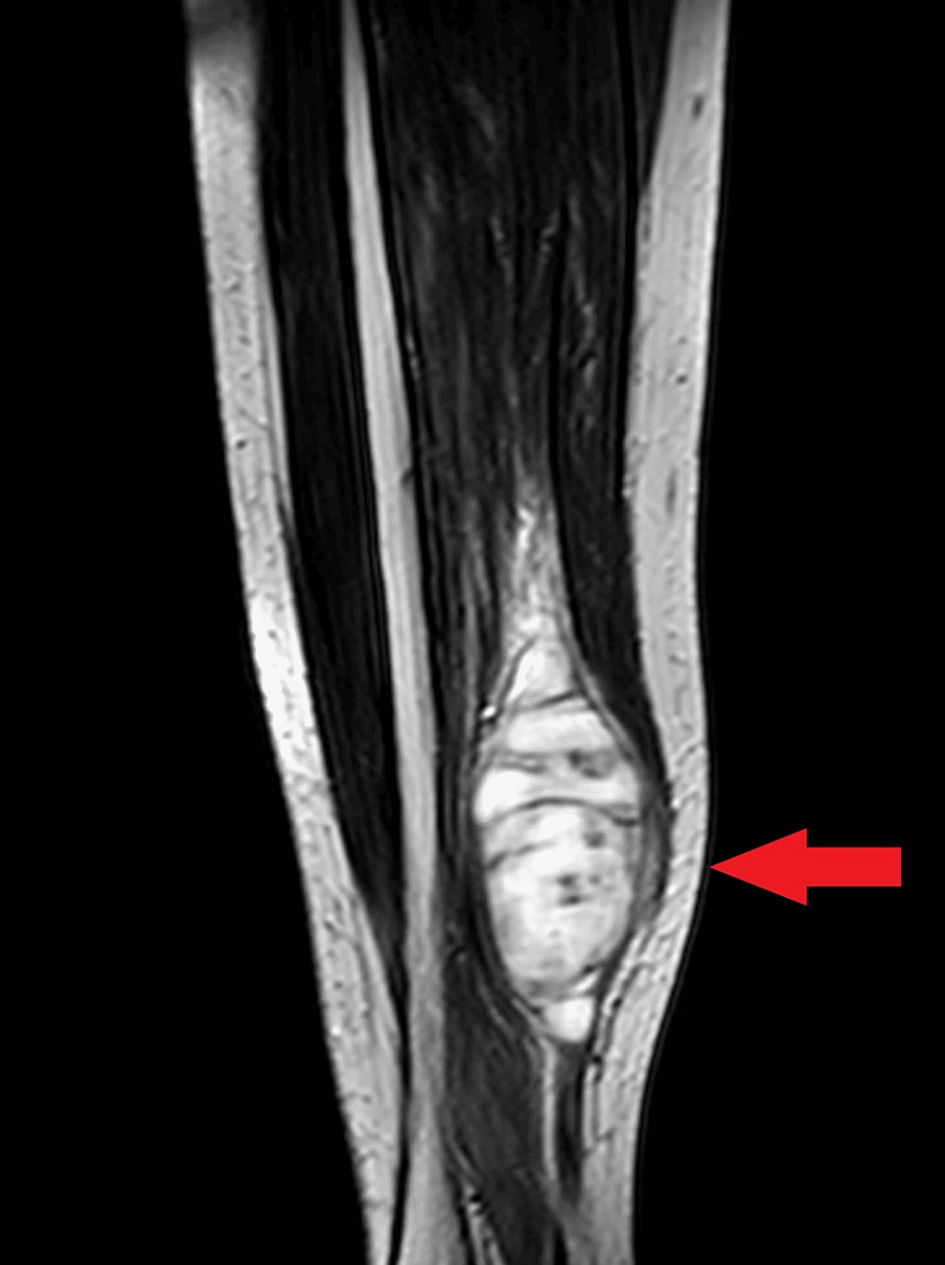
Contrast-enhanced MRI (coronal view) of the right leg showing a multi-lobulated spindle-shaped mass lesion with heterogeneous enhancement and altered signal intensity in the region of the knee joint

A tender, firm swelling with normal overlying skin was observed upon physical examination, prompting the physician to recommend an X-ray and blood investigation. The initial X-ray results did not yield a definitive diagnosis, as radiographic findings were within normal limits. Consequently, further diagnostic assessment was advised, leading to an MRI examination with contrast for the right leg. The MRI revealed a significant, multi-lobulated spindle-shaped mass lesion measuring 7.3 x 2.5 x 2.2 centimeters (Figure 1). The MRI evaluation included various sequences to provide comprehensive insights into the lesion. These sequences included T1-weighted, T2-weighted, and post-contrast T1-weighted sequences. On the T1-weighted sequence, the mass appeared hypointense, which can be characteristic of spindle cell sarcomas. In contrast, on the T2-weighted sequence, the lesion exhibited hyperintensity, suggesting differences in tissue composition. These variations in signal intensity between T1- and T2-weighted sequences can aid in the differential diagnosis of soft tissue tumors. Furthermore, the post-contrast T1-weighted sequence demonstrated heterogeneous enhancement within the mass, indicating variations in vascularity and tissue perfusion. In conjunction with altered signal intensity, this enhancement pattern is valuable for characterizing the tumor's behavior and is consistent with spindle cell sarcoma.

Diagnosing spindle cell sarcoma presents unique challenges, as this malignancy lacks specific imaging characteristics or clinical features that allow for a definitive diagnosis before histopathology. In our case, the diagnosis was established through clinical evaluation and diagnostic procedures. The initial clinical evaluation revealed a tender, firm swelling in the patient's right lower limb. While this presentation raised suspicion of possible malignancy, it was acknowledged that spindle cell sarcomas do not exhibit distinctive clinical features. Hence, the clinical evaluation alone was insufficient to make a precise diagnosis. However, it prompted further investigations.

A series of diagnostic procedures were initiated to characterize the lesion and confirm the diagnosis. MRI played a crucial role in visualizing the tumor's size, location, and features, providing insights that raised suspicion of a spindle cell sarcoma. It is important to note that while MRI findings can be suggestive, they are not specific for spindle cell sarcoma. Simultaneously, to alleviate the patient's symptoms, analgesics and antibiotics were prescribed as part of the initial management. These measures were taken to address pain and mitigate potential infection.

**Figure 2:**
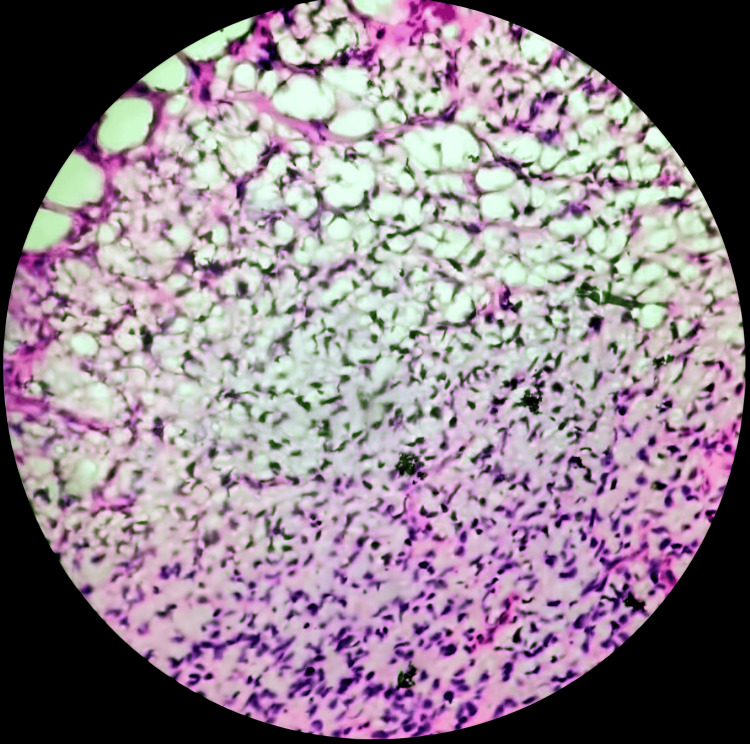
Histopathology slide showing spindle cell soft tissue sarcoma

However, recognizing the necessity of histopathological confirmation, a biopsy was strongly recommended. The biopsy procedure, carried out with precision, yielded a tissue specimen for histopathological examination. This examination was conducted under high magnification and meticulous scrutiny, which allowed for the definitive diagnosis of spindle cell soft tissue sarcoma (Figure 2). The histopathological assessment, including immunohistochemistry, enabled the characterization of spindle cell sarcoma based on tissue morphology and markers. The patient's case was discussed comprehensively with a multidisciplinary team regarding the management and treatment plan. Surgical resection was planned as the primary treatment modality. However, the specific surgical details, such as the extent of resection and adjuvant therapy, were determined based on the tumor's grade and location and in consideration of the patient's overall health.

## Discussion

Although rare, spindle cell soft tissue sarcomas pose significant diagnostic and therapeutic challenges. The case of this 70-year-old female patient highlights several important aspects of managing spindle cell sarcomas. The nonspecific clinical presentation of spindle cell sarcomas, such as pain and swelling, often leads to misdiagnosis or delayed diagnosis. This is not an isolated occurrence; similar diagnostic difficulties have been documented in the literature [12]. The ability to differentiate between benign and malignant conditions based solely on clinical presentation is frequently elusive. In the case of our patient, it's noteworthy that the swelling was painful and tender. While it is uncommon for sarcomas to elicit tenderness, various factors can contribute to this clinical observation. It is essential to consider potential causes for tenderness in such cases. Tenderness in soft tissue sarcomas can arise due to tumor location, pressure on adjacent structures, or necrotic areas within the mass. Spindle cell sarcomas, although typically associated with deep soft tissue, can sometimes develop near superficial structures, leading to tenderness upon palpation. In our patient, the location of the spindle cell sarcoma, its proximity to sensory nerves, or the presence of areas of necrosis within the tumor may have contributed to the tenderness. Detailed assessment and clinical examination are imperative to ascertain the underlying causes of tenderness in cases of spindle cell sarcomas. It is essential to understand that while tenderness is atypical in sarcomas, its presence should not preclude consideration of malignancy. Clinicians should remain vigilant and consider multiple clinical and imaging factors when evaluating such cases to ensure accurate and timely diagnosis [12].

Advanced imaging techniques play a crucial role in assessing spindle cell sarcomas. In the current case, MRI played a pivotal role in identifying the extent and characteristics of the tumor. This aligns with findings from previous studies that emphasize the role of MRI and CT in providing critical information for diagnosis and treatment planning [13,14]. The diagnostic journey in this case began with advanced imaging via MRI, which revealed a significant, multi-lobulated spindle-shaped mass lesion (Figure 1). The MRI evaluation included various sequences to provide comprehensive insights into the lesion. These sequences included T1-weighted, T2-weighted, and post-contrast T1-weighted sequences. On the T1-weighted sequence, the mass appeared hypointense, while on the T2-weighted sequence, it exhibited hyperintensity. The post-contrast T1-weighted sequence demonstrated heterogeneous enhancement within the mass, indicating variations in vascularity and tissue perfusion. Although advanced imaging strongly suggested a spindle cell sarcoma, it's important to note that the definitive diagnosis was subsequently confirmed by histopathological examination of the biopsy specimen. The biopsy, analyzed under high magnification, provided conclusive evidence of spindle cell soft tissue sarcoma, and this finding was consistent with established practice. This underscores the crucial role of expert pathologists in the diagnostic process [3].

Immunohistochemistry plays an essential role in identifying specific subtypes of spindle cell sarcomas. In our case, immunohistochemical staining was performed to characterize the tumor further. Notably, the marker CD34 was prominently expressed, underscoring its utility in aiding subtype classification [7]. CD34 is a valuable marker in diagnosing spindle cell sarcomas, and its presence can provide valuable insights into the tumor's nature and behavior. Other markers, such as S-100 and desmin, were evaluated to complement the immunohistochemical profile. While these markers did not show significant expression, their assessment is still important in ruling out certain subtypes and ensuring a comprehensive characterization. Treating spindle cell sarcomas typically involves surgical resection, as was performed in our case. The role of radiation therapy and chemotherapy depends on the tumor's grade and location [15]. Similar management strategies have been reported in the literature, reflecting the established standard of care for these malignancies. Furthermore, a multidisciplinary approach involving orthopedicians, radiologists, pathologists, and oncologists is essential for a comprehensive understanding of the disease and its optimal management [11]. Collaboration between these specialties was pivotal in achieving a diagnosis and formulating a treatment plan tailored to the specific subtype and characteristics of the spindle cell sarcoma in this case.

## Conclusions

In conclusion, the case report emphasizes the importance of considering spindle cell sarcomas in the differential diagnosis of patients with chronic limb pain and swelling. Timely recognition and a collaborative, interdisciplinary approach are essential for accurate diagnosis and appropriate treatment planning. Early intervention is paramount in improving the prognosis and quality of life for individuals affected by spindle cell soft tissue sarcomas. This case reinforces the significance of these considerations and underscores the need for continued research and clinical vigilance in managing this rare malignancy.
